# Complete childhood vaccination and associated factors among children aged 12–23 months in Dabat demographic and health survey site, Ethiopia, 2022

**DOI:** 10.1186/s12889-023-15681-0

**Published:** 2023-05-02

**Authors:** Abebaw Addis Gelagay, Abebaw Gebeyehu Worku, Debrework Tesgera Bashah, Nigusie Birhan Tebeje, Mignote Hailu Gebrie, Hedija Yenus Yeshita, Endeshaw Adimasu Cherkose, Birhanu Abera Ayana, Ayenew Molla Lakew, Desalegn Anmut Bitew, Desale Bihonegn Asmamaw, Wubshet Debebe Negash, Tadele Biresaw Belachew, Elsa Awoke Fentie

**Affiliations:** 1 Department of Reproductive Health, Institute of Public Health, College of Medicine and Health Sciences, University of Gondar, Gondar, Ethiopia; 2 School of Nursing, College of Medicine and Health Sciences, University of Gondar, Gondar, Ethiopia; 3 School of Midwifery, College of Medicine and Health Sciences, University of Gondar, Gondar, Ethiopia; 4 Department of Obstetrics and Gynecology, Zewuditu Memorial Hospital, Addis Ababa, Ethiopia; 5 Department of Epidemiology and Biostatistics, Institute of Public Health, College of Medicine and Health Sciences, University of Gondar, Gondar, Ethiopia; 6 Department of Health Systems and Policy, Institute of Public Health, College of Medicine and Health Sciences, University of Gondar, Gondar, Ethiopia

**Keywords:** Complete childhood vaccination, Children aged 12–23 months, Dabat demographic health survey site, Ethiopia

## Abstract

**Introduction:**

Childhood immunization is one of the most cost-effective public health strategies to prevent children’s mortality and morbidity from infectious diseases, but the Covid-19 pandemic and associated disruptions have strained health systems, and worldwide 25 million children missing out on vaccination in 2021. Of the 25 million, more than 60% of these children live in 10 countries including Ethiopia. Therefore, this study aimed to assess complete childhood vaccination coverage and associated factors in the Dabat district.

**Method:**

A community-based cross-sectional study was conducted from December 10/2020 to January 10/2021Gregorian Calendar. The data for this study was extracted from information collected for the assessment of maternal, Neonatal, and Child Health and health services utilization in the Dabat demographic and health survey site. Vaccine-related data were collected using an interviewer-administered questionnaire. An adjusted odds ratio with a 95% confidence interval was used to identify the presence and the direction of the association.

**Results:**

Based on vaccination cards and mothers/caretakers’ recall 30.9% (95%CI: 27.9-34.1%) of children aged 12–23 months in the Dabat district were completely immunized. Urban residency [AOR 1.813, 95% CI: (1.143, 2.878)], delivered in the health facility [AOR = 5.925, 95% CI: (3.680, 9.540)], ANC follow-up during their pregnancy [AOR 2.023, 95% CI: (1.352, 3.027)], rich wealth index [AOR = 2.392, 95% CI: (1.296, 4.415)], and parity [AOR 2.737, 95% CI: (1.664, 4.500)] were significantly associated with complete child vaccination.

**Recommendation and conclusion:**

Complete vaccination coverage among children aged 12–23 months in the Dabat district was lower than the Global vaccine plan and Ethiopian ministry of health goal in 2020. Therefore, Health care providers and other stakeholders should mobilize the community to improve mothers’ health-seeking behavior toward pregnancy follow-up and health facility delivery to improve childhood vaccination. Besides, expanding the service to remote areas are necessary to increase the immunization access.

## Introduction

Since the beginning of the Expanded Program on Immunization (EPI) by the World Health Organization (WHO), childhood immunization is one of the most cost-effective public health strategies to prevent children’s mortality and morbidity from infectious diseases, each year, it averts 2–3 million deaths of children [1]. The global number of deaths among children under the age of five dramatically decreased when the Expanded Program on Immunization (EPI) was launched, falling from 12.5 million in 1990 to 5.2 million in 2020 [2]. Sub-Saharan Africa (SSA), especially East Africa, continues to have the highest infant and under-five mortality rates in the world [3], and this could be closely linked to the low uptake of vaccinations.

The COVID-19 pandemic and associated disruptions have strained health systems and left 25 million children missing out on vaccination in 2021, of the 25 million, more than 60% of these children live in 10 countries, including Ethiopia. On the other hand, by the end of 2021, about 81% of infants worldwide (105 million infants) received 3 doses of the Diphtheria, Pertussis, and Tetanus (DPT3/ Penta-3) vaccine [4]. Although immunization coverage has improved globally, there is a significant disparity in the coverage of vaccines among countries: the performance of DPT1 and DPT3 in European countries is 97 and 94% respectively, whereas it is 84 and 76% for African countries [5], and for the measles vaccine, it is 95% in developed countries and 74% in Africa [6].

The 2016 Ethiopian Demographic and Health Survey (EDHS) report revealed that only 39% of children in Ethiopia received all recommended immunizations [7]. Additionally, according to the 2019 mini-EDHS report, full immunization coverage has reached 43%, with a steady change over time [8]. There is a wide variation of coverage of full immunization among regions of Ethiopia, is highest in Addis Ababa (89%), Dire Dawa (76%), and Tigray (67%) and lowest in Affar (15%), Somali (22%), and Oromia (25%) [7].

Different factors can play a role in completing routine childhood immunizations. These include urban residence, mother’/caregiver’s age, educational status of the mother and father of the child, maternal occupation status, marital status of the mother/caregiver, wealth status, distance from the health facility, low access to the service, inconvenient immunization schedule, media exposure, mothers who have good knowledge on vaccination, parity, place of delivery, ANC follow-up during pregnancy, tetanus toxoid (TT) vaccination of the mother during pregnancy, and postnatal care (PNC) follow- up [9–17].

The long-term goal of the Ministry of Health EPI Strategy is to achieve 90% DPT3 coverage in all regions by reaching every district [18]. So, EPI in Ethiopia provides immunization services through routine, outreach, and mobile sites to the target groups residing in every corner of the country. Despite these huge efforts made over decades by the federal ministry of health (FMoH) along with its partners a large number of children have not been immunized. Therefore, this study aimed to assess complete vaccination coverage and associated factors in the Dabat district.

## Method

### Study design and period

This is a community-based cross-sectional survey conducted in the Dabat demographic and health survey site (DHSS) from December 10/2020 to January 10/2021 Gregorian calendar (GC).

### Study area

Dabat town is the capital of the Dabat district, which is about 76 km away from Gondar town (Zonal town) to the north. According to the Dabat woreda health office report, the projected estimate of the population in the district was 189,944 in the 2020/2021 Gregorian calendar (GC). There was a total of 44,789 women of reproductive age, 25,718 under-five children, and 6,401 infants. The Dabat woreda has a total of 36 kebeles (smallest administrative units in Ethiopia) of which 31 are rural. In the district, there are 6 health centers and 29 health posts. The Dabat demographic and health survey (DHS) is one of the six health and demographic surveillance systems in Ethiopia. The DHSS consists of 13 Kebeles (9 rural and 4 urban).

### Source and study population

The source population was all children aged 12 to 23 months with their mothers or caretakers living in the Dabat district. The study population was those children aged 12 to 23 months with their caregivers living in Dabat demographic and Health Survey sites.

### Eligibility criteria

Mothers or caregivers who had an alive child aged between 12 and 23 months, resided in the Dabat district and could bring a vaccination card or recall vaccination history were included in the study.

### Sample size determination and sampling procedure

The required sample size was determined by using the single population proportion formula Z_α/2_^2^ *p*(1-p) / d^2^ .considering an estimated 75.1% prevalence of complete vaccination among children aged 12–23 months from a similar study conducted in Gondar city administration [19]. In addition, by taking the margin of error (d) as 3% and 95% confidence interval, and adding a 10% non-response rate. Thus, the calculated final sample was 880. However, the predicted sample size is larger than the data collected for the project on maternal, neonatal, and child health (MNCH) and health services utilization in Dabat Demography Health Surveillance Systems. Therefore, all the data collected from eligible participants for this study were included in the analysis.

University of Gondar, Institute of Public Health selects Dabat Demographic and Health Survey sites to represent each of the district’s agroecological zones. This study applied census method to identify and select eligible study participants.

### Study variable

#### Dependent variable

Complete vaccination (yes/no).

#### Independent variable

Socio-demographic characteristics of the mothers/ caregiver (age of the mother, residency, educational status, occupational status, family size, and wealth index), children characteristics (sex of the child, age of the child. Birth order, and place of delivery), obstetric and health service-related characteristics of the mother (parity, ANC follow up, TT vaccination, and PNC follow up) were considered as independent variables for this study.

### Operational definition

#### Complete vaccination

was defined as an infant has received all recommended vaccines included in the national schedule: a dose of Bacille Calmette Guérin (BCG); three doses of Oral Polio Vaccine (OPV); three doses each of Penta-valent and Pneumococcal Conjugate Vaccine (PCV); one dose of.

Inactivated Polio Vaccine (IPV); two doses of rotavirus and one dose of measles vaccines by the age of 12 months [16].

### Data Collection tools and procedures

The questionnaire was developed in English from related literature and translated to Amharic, the local language of the area, and then translated back to English for consistency and analysis. A structured and pretested questionnaire was used to collect the data. The data were collected through face-to-face interviews with mothers or caregivers of the child. Vaccination status was determined by obtaining vaccination cards from the mother or caregiver, vaccination history from the mother or caregiver, or both. If the child has a vaccination card, we asked that her to bring it, and data collectors must record all of the vaccines the child received according to the EPI schedule. Other probing questions and techniques were considered to ensure that the child has had all recommended vaccinations if there’s no vaccination card for a different reason. For instance, for the BCG vaccine, the mothers or caregivers were asked that their children were given an injection in the right shoulder and observe a BCG scar in the right arm. For the polio vaccine, the mother or caregiver was asked, “has the child been given a polio vaccine that was dropped in the mouth?” If she said yes, “how many times was the polio vaccine administered? ” Similarly, for pentavalent vaccines, the mother or caregiver was asked, “Was the child given an injection given on the right thigh or buttocks?” If she replied “yes,” how many times was the child given? In addition, for measles, the mother or caregiver was asked, “was the child given the vaccine in the thigh or buttocks at age of 9 months?” [20].

Supervisors and enumerators from the research center were recruited and to ensure data quality, a five-day training was given for supervisors and enumerators about the study objectives and briefed on the content of the questionnaire and procedure before fieldwork.

### Data management and analysis

All questionnaires were checked for consistency and completeness. The data were entered into the computer using Epi-data version 3.1 and exported to the statistical package for social science (SPSS) version 25. First, the data were cleaned and coded. Descriptive analysis was performed to generate the frequency and proportion of dependent and socio-demographic variables. Bivariable and multivariable logistic regression analyses were done to determine the presence of an association between dependent and independent variables. In bivariable logistic regression, those variables with a P-value less than 0.2 were entered into multivariable logistic regression analyses. In multivariable logistic regression analyses, variables with a P-value, of less than 0.05 were considered significant. The Hosmer–Lemeshow goodness of fit test was used to check the model’s fitness. An adjusted odds ratio with a 95% confidence interval was used to determine the presence and direction of association between covariates and the outcome variable.

## Result

### Sociodemographic characteristics of mothers/ caregivers

A total of 857 mothers with 12–23 months old children participated in this study. More than half of mothers (67.8%) were in the age range of 25–34 years, with a mean age of 30.80 ± 6.38. About, 62.2% of respondents reside in rural kebele. Regarding educational status, the majority (61.0%) of mothers or caregivers had no formal education, while 20.2% had secondary and above educational status. 816 (95.2%) of mothers or caregivers were married, 706 (82.8%) were housewives by occupation, and 212(24.7%) of respondents were poor wealth status (Table 1).

**Table 1:** Socio-demographic characteristics of mothers/ caregivers in DDHSS, 2022 (N = 857)

Variable	Category	Frequency	%
**Age of Mother/ caregiver**	15–19	13	1.52
	20–34	581	67.79
	≥ 35	263	30.69
Residency	Rural	533	62.19
	Urban	324	37.81
**Marital status**	Married	816	95.23
	Unmarried	41	4.78
Mother/ caregiver educational status	No formal education	523	61.03
	Primary	161	18.79
	Secondary & above	173	20.18
Mother/ caregiver occupational status	Housewife	708	82.61
	Employee (gov’t or private)	70	8.17
	other*	79	9.22
father’s educational status	No formal education	478	55.78
	Primary	206	24.04
	Secondary & above	173	20.18
father’s occupational status	Farmer	634	73.98
	Employee (gov’t or private)	110	12.84
	Other**	113	13.18
Family size	2–3	108	12.60
	4	272	31.74
	5	212	24.74
	≥ 6	265	30.92
Wealth index	Poorest	143	16.69
	Poor	212	24.74
	Middle	183	21.35
	Rich	142	16.57
	Richest	177	20.69

Other*= farmers, Daily laborers, and merchants; other**: Daily laborers and merchants; family size include mothers or caregivers;

### Obstetric and health service-related characteristics of mothers/ caregivers

More than half (58.1%) of the mothers or caregivers had antenatal care (ANC) follow-ups during their pregnancy. Almost half (49.8%)of them had taken one or more doses of the tetanus toxoid (TT) vaccine. Of the total respondents, 302 (35.2%) had postnatal care (PNC) follow-up (Table 2).

### Characteristics of 12-23 months children

Of the total of 857 children, 431 (50.3%) were females. 364 (42.5%) of children were in the age group 20-23 months, with a mean age of 18.15 ± 3.91. More than half of (58.9%) children were born at a health institution. Of the children included in the study, 417 (48.7%) were in the fourth or higher birth order (Table 3).

**Table 2:** Selected characteristics of 12–23 months children in DDHSS, 2022 (N = 857)

Variable	Category	Frequency	%
**Sex of child**	Male	426	49.71
	Female	431	50.29
**Age of child in months**	12–15	255	29.75
	16–19	238	27.80
	20–23	364	42.45
**Birth order**	First	130	15.17
	2–3	310	36.17
	≥ 4	417	48.66
**Place of delivery**	Home	352	41.07
	Health facility	505	58.930

**Table 3:** obstetric and health service-related characteristics of Mothers/caregivers in DDHSS, 2022 (N = 857)

Variable	Category	Frequency	%
Parity	Primipara	128	14.94
	Multipara	644	75.12
	Grand Multipara	85	9.94
ANC use	No	359	41.89
	Yes	498	58.11
TT vaccination of the mother	No	430	50.18
	Yes	427	49.82
PNC use	No	555	64.76
	Yes	302	35.24

ANC: Antenatal Care; TT : Tetanus Toxoid; PNC: Postnatal Care; Primipara: a woman who is giving birth for the first time; Multipara: a woman that has had more than one birth (live or stillbirth) > 28 weeks of gestation; Grand Multipara: a woman that has had ≥ 5 births (live or stillbirth) > 28 weeks of gestation

### Vaccine coverage for children

**Fig. 1:**
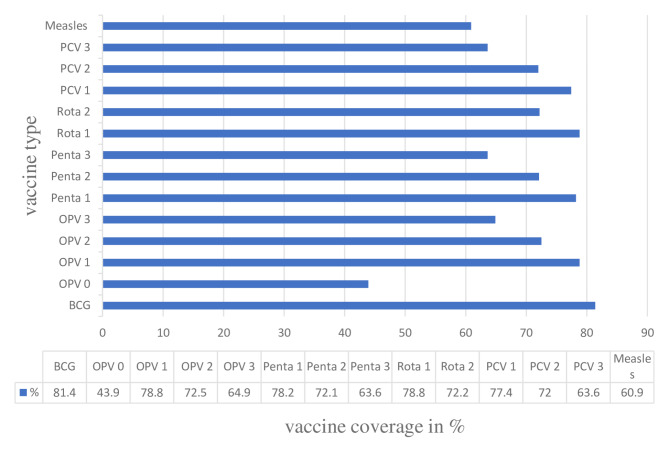
Vaccination coverage of 12–23 months old children in Dabat district, North west Ethiopia, 2021/2022

The total proportion of children who received all the required vaccines was 30.9% (95% CI: 27.9, 34.1). At the level of antigen-specific coverage, BCG was taken by the majority of children (81.4%), followed by OPV-1 (78.8%) and Rota-1 (78.8%). OPV-0 (43.9%) was the least taken vaccine compared with other vaccines (Fig. 1).

### Factors associated with complete child vaccination

On the bivariable analysis, residency, educational status of mother and husband, family size, wealth index, parity, antenatal care follow-up, having tetanus toxoid immunization, place of delivery, and postnatal care were found to be significantly associated with complete vaccination status with a P value of 0.2 and below. In multivariable analysis, residency, wealth index, parity, ANC follow-up, and place of delivery were found to be significantly associated with the complete vaccination with a P value of 0.05 and below.

Children born to mothers who reside in urban areas were 1.8 times more likely to complete their vaccination compared to those born to rural mothers [AOR 1.813, 95% CI: 1.143, 2.878]. Children delivered in the health facility were 5.9 times more likely to complete their vaccination than those delivered at home [AOR = 5.925, 95% CI: 3.680, 9.540]. Mothers who had ANC follow-up during their pregnancy were 2 times more likely to have complete vaccination of their children compared to mothers who did not have ANC follow-up during their pregnancy [AOR 2.023, 95% CI: 1.352, 3.027). Mothers or caregivers from rich households had a 2.4 times higher chance of completing their child’s vaccination (AOR = 2.392, 95% CI: 1.296, 4.415) as compared to mothers or caregivers from the poorest households. Furthermore, the odds of complete vaccination were 2.7(AOR = 2.737, 95% CI: 1.664, 4.500) times higher in multiparous women than in primiparous women (Table 4).

**Table 4:** Binary logistic regression analysis of factors associated with complete child vaccination in Dabat district demographic health survey site, North west Ethiopia, 2021/2022

Variables	Category	complete vaccination		COR(95%CI)	AOR (95% CI)
		No	Yes		
Residency	Rural	383	150	1	1
	Urban	209	115	1.41(1.05, 1.89)	1.81(1.14, 2.89) *
Mother/ caregiver educational status	No formal education	373	150	1	1
	Primary	106	55	1.29(0.89, 1.88)	0.88 (0.55, 1.42)
	Secondary & above	113	60	1.32(0.92, 1.90)	0.93 (0.51, 1.70)
Father educational status	No formal education	335	143	1	1
	Primary	146	60	0.96(0.67,1.38)	1.12 (0.66,1.89)
	Secondary & above	111	62	1.31(0.91,1.89)	0.74 (0. 0.45,1.33)
Family size	2–3	82	26	0.88(0.53,1.48)	1.27 (0.55, 2.97)
	4	180	92	1.42(0.98,2.06)	1.09 (0.57, 2.11)
	5	135	77	1.59(1.08,2.35)	1.27 (0. 0.81, 1.99)
	≥ 6	195	70	1	1
Wealth index	Poorest	107	36	1	1
	Poor	157	55	1.04(0.64, 1.69)	1.51 (0.88, 2.67)
	Middle	119	64	1.60(0.99, 2.60)	2.613 (1.46, 4.69) **
	Rich	84	58	2.05(1.24, 3.40)	2.39 (1.30, 4.42) *
	Richest	125	52	1.24(0.75, 2.03)	1.15 (0.59, 2.23)
Parity	Primiparity	102	26	1	1
	Multiparity	423	221	2.05(1.29,3.25)	2.74 (1.66, 4.50) ***
	Grand multiparity	67	18	1.05(0.54,2.07)	1.64 (0.77, 3.49)
ANC follow up	No	291	68	1	1
	Yes	301	197	2.80(2.04,3.85)	2.02 (1.35, 3.02) **
TT vaccination	No	332	98	1	1
	Yes	260	167	2.18(1.62, 2.93)	0.97 (0.56, 1.68)
Place of delivery	Home	301	51	1	1
	Health facility	291	214	4.34(3.07,6.13)	5.93 (3.68, 9.54) ***
PNC follow up	No	402	153	1	1
	Yes	190	112	1.55(1.15,2.09)	0.69 (0.47, 1.01)

*** P-value < 0.001, ** p-value < 0.01, *p-value < 0.05. Hosmer-lemshow value = 0.91

## Discussion

This study aimed to assess complete vaccine coverage and associated factors among children aged between 12 and 23 months living in the Dabat district. The complete vaccination status of the children was confirmed using their vaccination cards and vaccination history from the mother/ caregivers. This study found that the level of complete vaccination coverage in the study area was 30.9% (95% CI: 27.9, 34.1). This figure was higher compared with Afar (15%), Somali (22%), and Oromia (25%) [7]. This might be due to nomadic and pastoralist inhabitants in the Afar and Somali regions making it difficult to provide immunization services for all and those regions having weak healthcare systems, which led to low uptake of vaccines [21].

The finding of this study is also higher than studies done in Somalia (11.6%), Chad (11.4%), and the Republic of Central Africa (17.3%) [22]. The possible explanations might be due to the presence of differences in the health system and policies against immunization services, variability in the awareness of immunization services, and socio-cultural differences across countries [23].

However, the finding of this study is lower than studies done in Minjar Shenkora district, Ethiopia(75.6%) [24], Mizan Aman town (42.2%) [25], in Gondar city administration of Northwest Ethiopia (75.5%) [19], Haremaya District, Eastern Ethiopia (50.6%) [26], Woldiya, Ethiopia (87.7%) [27], East Africa (69.21%) [12] Benin (85.5%) [28], Nigeria (58%) [29], and Ghana (89.5%) [30]. These disparities could be due to differences in the availability and accessibility of immunization services [26]. Moreover, the discrepancy might be due to this study was done after the onset of the COVID-19 pandemic, and during the times of quarantine or early stage of the COVID-19 pandemic, vaccination activities in all age groups, especially routine vaccines for children, were interrupted, delayed, or completely transferred to another time, which leads to decline childhood vaccination rates [31].

The coverage showed that there is a decrement from the first dose of OPV, PCV, and Pentavalent to the last dose. There is a 20%-point dropout rate from BCG to measles vaccine, a 14.6% dropout rate from Penta 1 to Penta 3, and a 13.9%-point dropout rate from the first to the third dose of polio vaccine. This might be related to vaccine hesitancy due to cultural misconceptions, adverse effects of vaccination, and poor management of adverse effects of vaccination [12].

In this study, urban children were more likely to complete their vaccinations compared to their rural counterparts. This finding is supported by studies done in Lay Armachiho District [32], Mecha District, Northwest Ethiopia [10], Ethiopia [21], and Maynamar [33]. The possible explanation might be low awareness, lack of access to health facilities, and poor health-seeking behavior of rural residents including vaccination of their children [21].

Another factor that affects complete vaccination is ANC follow-up during pregnancy. children whose mothers had antenatal care (ANC) follow-ups were more likely to be fully vaccinated than those who did not attend ANC. This finding is supported by studies done in Brazil [34], Nigeria [29], Democratic Republic of Congo [35], East Africa [12], Mizan Aman Town [25], and Sekota Zuria District [36]. This might be due to mothers, during ANC visits, receiving counseling and education about the importance of child vaccination, which results in higher compliance with the recommended immunization schedule [37].

Furthermore, the findings of this study revealed that the odds of complete vaccination were 2.7 times higher in multiparous women than in primiparous women. This finding is in line with studies done in Benin [28], Brazil [34], and East Africa [12]. It could be due to multiparous women know about child health services, including immunization services, from their previous experience, which may have a positive influence on the acceptance and compliance of complete vaccination.

Regarding the place of delivery, children delivered in a health facility were 5.9 times more likely to complete their vaccination than those delivered at home. This finding is consistent with studies done in Senegal [25], Benin [28], Sub-Saharan Africa [23], Mecha District [10], and Jijiga District [11]. This might be due to delivery in the health institution creating an opportunity to vaccinate BCG and OPV 0 at birth [20], or it may create an opportunity to discuss with health professionals the importance of immunization, the timing of vaccine initiation, when a vaccine is completed, and possible side effects associated with the vaccine, which results in higher compliance towards the recommended immunization schedule [21].

Children from medium- and high-wealth households were more likely to complete their vaccination than children from poor households. this finding is in line with studies done in Maynamar [33], Malawi [38], Democratic Republic of Congo [35], sub-Saharan Africa [23], and Ethiopia [21]. The possible explanation might be due to wealthier people have healthier childcare practices and better health-seeking behavior, or travel cost to health facilities can restrict poor people’s willingness to immunize their children [12].

Despite generating this important evidence, our study had limitations. In addition to reviewing vaccination cards, information about the basic vaccinations was collected based on mothers’ verbal responses. This might cause a lot of recall bias, which leads to overestimating or underestimating the coverage.

## Conclusion and recommendation

Complete vaccination coverage among children aged 12–23 months in the Dabat district was lower than the national coverage and the Global Vaccine plan. Residence in the urban area, ANC follow-up, parity, place of delivery, and economic status of the household were significantly associated with the complete vaccination status of children in the district. Therefore, healthcare providers and other stakeholders should mobilize the community to improve mothers’ health-seeking behavior toward pregnancy follow-up and health facility delivery to improve childhood vaccination. Besides, expanding the service to remote areas is necessary to step up immunization access.

## Data Availability

All relevant data are within the manuscript.

## Abbreviations

ANC: Antenatal care
AOR: Adjusted Odd Ratio
BCG: Bacilli Calamite Guerin
CI: Confidence Interval
COR: Crude Odds Ratio
DDHS: Dabat Demographic and Health Survey Site
DPT: Diphtheria, pertussis, and tetanus
EDHS: Ethiopia Demography Health Survey
EPI: Expanded program of immunization
OPV: Oral polio vaccine
PCV: Pneumococcal conjugate vaccine
WHO: World Health Organization
